# Fat‐tailed dispersal shapes connectivity in a solitary bee

**DOI:** 10.1002/ecy.70437

**Published:** 2026-07-22

**Authors:** Nicholas N. Dorian, Elizabeth E. Crone

**Affiliations:** ^1^ Program in Plant Biology & Conservation Northwestern University Evanston Illinois USA; ^2^ Negaunee Institute for Plant Conservation Science and Action Glencoe Illinois USA; ^3^ Department of Biology Tufts University Medford Massachusetts USA; ^4^ Department of Evolution & Ecology University of California Davis California USA; ^5^ Present address: Department of Biology, City College of New York City University of New York New York New York USA

**Keywords:** central‐place foraging, *Colletes validus*, dispersal kernel, habitat connectivity, long‐distance dispersal, natal dispersal, pollinator conservation

## Abstract

Dispersal—movement potentially leading to gene flow—is a fundamental ecological process yet notoriously difficult to measure. For solitary bees in particular, determining the spatial scale of dispersal movements and how landscape features influence connectivity is key to achieving conservation goals. Over 2 years, we marked 1698 females of the solitary bee *Colletes validus* at nesting sites, recaptured marked bees as they dispersed to build nests and as they foraged on flowers, and fit statistical kernels to observed movement distances. In total, we observed 349 dispersal events by 313 individual bees including 13 natal dispersal events (movement from emergence to the first nesting site) and 336 renesting dispersal events (movement among subsequent nesting sites). Solitary bee dispersal kernels were fat‐tailed: Most bees made local movements (mean = 143 m), but a small proportion in the distributional tails made substantially longer movements (99% kernel quantile = 1.2 km) and the farthest dispersers were expected to move nearly 3 km. We also recorded 38 foraging movements from nests to flowers by 38 individual bees, allowing us to compare two ecologically distinct movement types. Mean foraging (131 m) and dispersal distances were similar, but upper foraging distances (99% kernel quantile = 554 m) were substantially lower than dispersal. Movement patterns also depended on landscape composition: Both foraging and dispersal movement propensity decreased with increasing inter‐patch distance and number of road crossings. Our study highlights fundamental differences in the tails of foraging and dispersal in a central‐place forager, demonstrates how landscape features constrain solitary bee movements, and contributes quantitative metrics on the spatial scale of movement for pollinator conservation planning.

## INTRODUCTION

Dispersal—broadly defined as movement potentially leading to gene flow—is a fundamental driver of ecological and evolutionary processes such as habitat selection, persistence in patchy landscapes, and range expansion in changing environments (Kot et al., 1996; McConkey et al., 2012; Travis et al., 2013). For conservation, accurate estimates of the frequency and scale of dispersal movements are critical, particularly when site colonization and patch connectivity are priorities (Driscoll et al., 2014; Leidner & Haddad, 2011). However, characterizing dispersal and its influence on ecological processes is notoriously difficult, in part because the distributional tails comprise rare, hard‐to‐observe movements.

The challenge of measuring dispersal is particularly acute for central‐place foragers like seabirds, rodents, and bees, which are animals that return food to a fixed location (Friedlaender et al., 2016; Olsson et al., 2008). In these taxa, dispersal and foraging are distinct movement processes: Foraging movements occur repeatedly from nests to food sources during the breeding season, whereas dispersal movements among breeding sites occur infrequently throughout an individual's life. Although dispersal in central‐place foragers has been well studied in vertebrates (Driscoll et al., 2014), solitary bees possess traits that also make them appealing study systems. Female solitary bees—which live in nests built by a single female, rather than by a queen and workers—can create multiple nests during their short lifetimes (Danforth et al., 2019). This means that multiple dispersal events in the forms of natal dispersal (movement from emergence site to first nest) and renesting dispersal (movements among successive nests built by the same individual) occur over weeks rather than years. In addition, because nesting sites are often clustered over hundreds of meters (Bischoff, 2003; Franzén & Nilsson, 2013), dispersal movements in solitary bees occur over spatial scales that are more tractable for field studies than in many vertebrate animals.

Despite the potential suitability of solitary bees as model systems for studies of movement ecology, our understanding of solitary bee dispersal is limited. To date, a handful of studies have documented local, natal dispersal movements of solitary bees within a single nesting site. For example, 50% of digger bees *Diadasina distincta* exhibited a natal dispersal distance of less than 5 m (Antonini et al., 2000), and 80% of mason bees *Osmia rufa* built nests in the same nesting block from which they emerged (Steffan‐Dewenter & Schiele, 2004). These local‐scale observations, however, contrast with evidence of solitary bee dispersal over much larger distances. Records of capture offshore (Murray et al., 2009), rapid range expansion (Gibbs & Sheffield, 2009), and low genetic differentiation over tens to hundreds of kilometers (Cerná et al., 2013; Exeler et al., 2008) imply that the maximum dispersal distances of solitary bees might be great, but to date they remain unquantified. Such a finding would not be unexpected: In many non‐bee taxa, the dispersal distribution is fat‐tailed or leptokurtic (Bullock et al., 2017; Fandos et al., 2023), meaning that most individuals make local movements, but a few individuals exhibit extreme long‐distance movements (Nathan, 2001). Although rare, these long‐distance movements can disproportionately drive ecological dynamics such as site colonization, metapopulation persistence, and range shifts (Sullivan et al., 2021; Trakhtenbrot et al., 2005). Leptokurtic dispersal processes typically arise from a combination of factors including variation in movement behavior among individuals, and the presence of multiple dispersal behaviors within a single population (e.g., short‐distance philopatry coupled with long‐distance movements). Given that both local and long‐distance movements have been reported for bees, we hypothesize that solitary bee dispersal is leptokurtic, a pattern that would reconcile the apparent discrepancy between data across scales.

Here, we present the first comprehensive estimates of dispersal for a solitary bee. Over 2 years, we paint‐marked individual *Colletes validus* at emergence and nesting sites, tracked nest‐building bees to measure both natal and renesting dispersal distances, and fit statistical kernels to observed movement distances to characterize the entire dispersal distribution. We also measured foraging movements from nest sites to flowers and fit statistical kernels to the distribution of foraging distances, allowing us to compare two ecologically distinct movement types within the same system. Last, since landscape composition and conspecific presence can influence movement patterns, we tested the influence of four factors on bee movement: (1) distance among patches, (2) number of road crosses, (3) population density at origin patch, and (4) destination patch area. Beyond advancing a basic understanding of the movement ecology of central‐place foraging insects, our study advances restoration of functionally connected populations of wild pollinators (Schultz et al., 2018). Nearly 70% of the 4000 species of wild bees in North America are solitary, and many are thought to be declining (Danforth et al., 2019); yet missing information on solitary bee dispersal in natural systems hampers effective conservation planning (U.S. Fish and Wildlife Service, 2022). By providing the first quantitative benchmarks of solitary bee dispersal, our study clarifies the spatial scale at which populations are connected by movement and demonstrates how population connectivity can be shaped by landscape features.

## METHODS

### Study system

**FIGURE 1:**
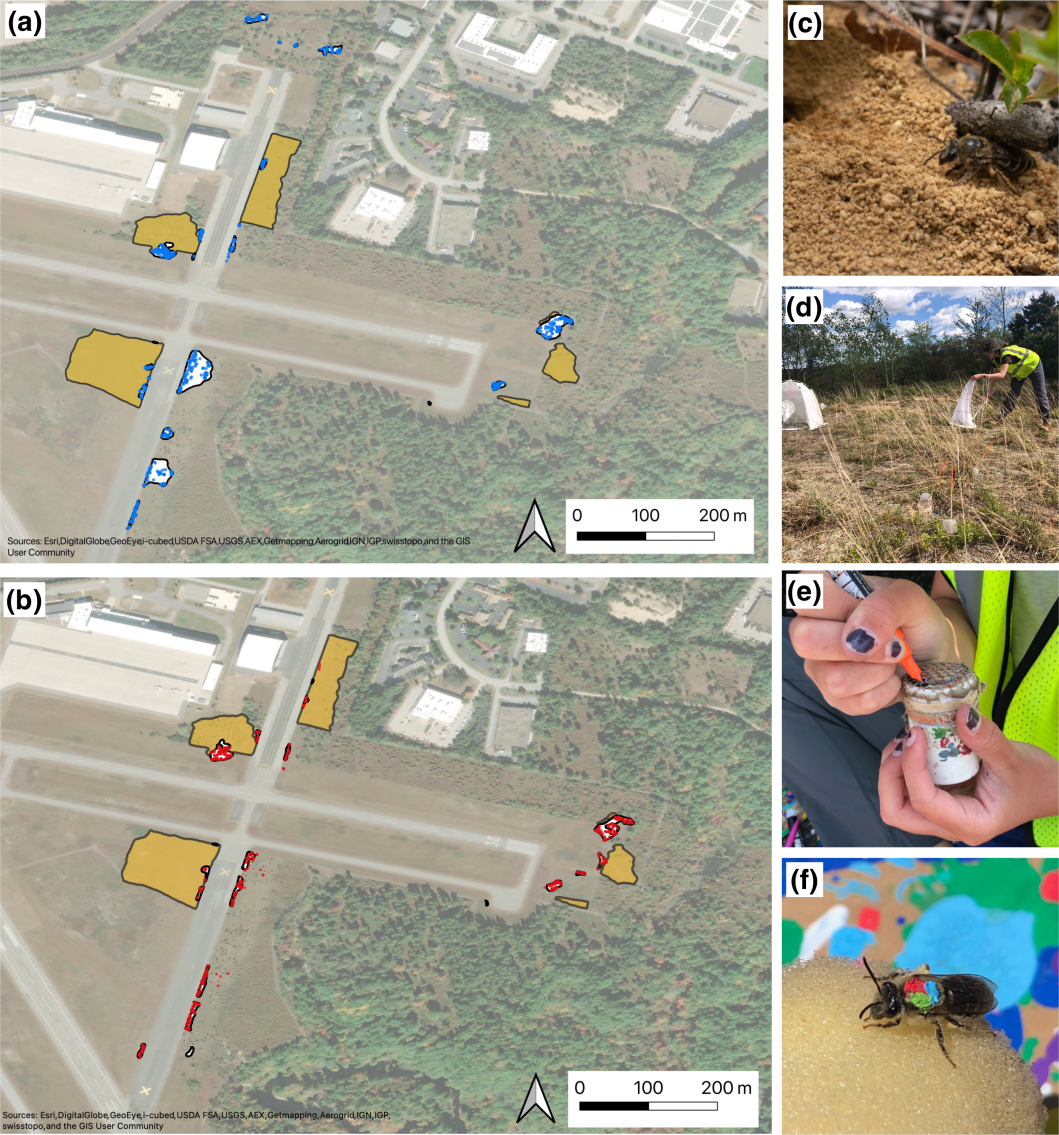
We measured solitary bee dispersal and foraging movements at Concord Airport, New Hampshire. Nesting aggregations, nests, and foraging survey areas were mapped in (a) 2021 and (b) 2022. Colored points denote nest locations, white polygons denote aggregations, and yellow polygons denote foraging survey areas. Maps created by Nicholas N. Dorian using icons from QGIS. Basemap source: Esri, DigitalGlobe, GeoEye, i‐cubed, USDA FSA, USGS, AEX, Getmapping, Aerogrid, IGN, IGP, swisstopo, and the GIS User Community. (c) We located active *Colletes validus* nests in nesting aggregations. (d) Nesting females were captured in emergence tents or at their nests. (e) Captured females were individually marked on their thorax using three colors of paint. (f) A marked female *C. validus*. Photographs in (c–f) taken by Nicholas N. Dorian.

The blueberry cellophane bee *Colletes validus* Cresson (Hymenoptera: Colletidae) is a solitary, ground‐nesting bee that occurs in pine barrens in eastern North America. At our study site, bees emerge in late April and mate, and females may disperse to build their first nest (hereafter, natal dispersal). Females typically build nests in sandy, sparsely vegetated soils in aggregations (1–10 nests‐m^−2^) (Figure 1). Females make daily foraging trips to gather nest provisions (pollen and nectar) predominately from *Vaccinium* spp. flowers (Batra, 1980; Dorian, 2024). Upon completing their first nest, some females disperse one or more times to build and provision subsequent nests (hereafter, renesting dispersal). To our knowledge, females construct only one nest at a time.

### Movement surveys

We studied *C. validus* movement at Concord Airport, Concord, New Hampshire (~50‐ha site area, 43.206° N, 71.509° W), from April to June 2021 and 2022. Throughout the flight season, we conducted mark–recapture surveys for females at nesting sites to track dispersal and at flowers to track foraging. Males were not included in our study since they do not forage for pollen or construct nests, so their movements are likely characterized by different patterns than females, and for the practical reason that they were absent from aggregations after the first week of the nesting season. Surveys took place on sunny, warm, and calm days (>10°C, <9 m/s wind).

We measured dispersal via mark–recapture surveys at nesting aggregations. Surveys occurred across 17 aggregations in 2021 (mean distance between aggregation centroids = 308.7 m, range = 11.0–848.2 m) and across 21 nesting aggregations in 2022 (mean distance between aggregation centroids = 356.6 m, range = 9.3–947.6 m) (Figure 1). Aggregations were located by repeatedly searching across the airport grounds for tumuli (i.e., nest entrances) indicating an active nesting site. Aggregations contained between 10 and 500 active nests on any given day. We looked for new nests by searching a grid within the aggregation and along the aggregation perimeter 10 paces away from the closest nest. In most cases, it was straightforward to delineate the edge of an aggregation since either suitable nesting habitat disappeared, for example, vegetation abruptly transitioned to asphalt, sparsely vegetated areas transitioned to oak‐pine scrub or *Vaccinium* heath, or the density of nests conspicuously dropped off.

To study natal dispersal, we monitored emergence of females from known nest locations. We placed emergence tents (0.5 m × 0.5 m mesh tents; BugDorm, Taiwan) over nests marked during the prior year. Tents were deployed over clusters of nests because high nesting density made it impossible to monitor just one nest at a time. In 2021, we monitored 142 nests from 50 clusters across 8 nesting aggregations, and in 2022, we monitored 83 nests from 40 clusters across 10 aggregations. During each survey, we checked emergence tents for any bees that had emerged since the prior survey and then captured and paint‐marked newly emerged females (as described below). Tents remained over the nests for the duration of the emergence period and were checked daily for emergence (May 2–14 in 2021 and April 25–May 16 in 2022).

To monitor movement between nests, we searched for and marked all active nests with uniquely numbered tags at each aggregation, that is, all active nests on the first survey, and all new, active nests on subsequent surveys. Nest searching ceased when no new nests were found after 10 min of searching by one or two observers. Then, small plastic cups were placed over all open nests (Figure 1d). For 45 min, we netted all bees departing or attempting to enter a cupped nest. Small, dense aggregations were surveyed by one person who could realistically watch all covered nests simultaneously; Larger, diffuse aggregations were surveyed by two people to ensure that all nests received approximately equal observation effort. The largest aggregations were subdivided into sampling units, each of which was monitored by two individuals for separate 45‐min periods. We also caught bees engaging in nest‐searching behavior defined as slow flights close to the ground, which we scored as nesting attempts within the aggregation. For every bee captured, we recorded the date, time, aggregation, and nest number. Previously marked bees were recorded as resights and released. Unmarked bees were painted with unique combinations of three colored, nontoxic paints on the thorax (Posca USA; Figure 1e,f). Paint marking typically took less than 1 min. We surveyed active aggregations every other day for nesting females (2021: May 12–June 2 and 2022: May 11–June 4). Aggregations were visited between two and seven times depending on the duration of nesting activity. At the end of the flight season, geographic coordinates (±0.5 m) of emergence traps and nest locations, and polygons of aggregations, were collected using Trimble Geo7x.

We studied foraging movements by searching for previously marked individuals on flowers. We established four foraging survey areas which were adjacent to nesting aggregations and which contained flowering *Vaccinium* spp. (Figure 1a,b). We conducted foraging surveys between 8 a.m. and 12 p.m. by searching a grid across each survey area for marked bees for 45 min. Upon locating a patch of flowers within a survey area, we scanned for marked bees, and if none were observed, moved on to the next patch within the survey area. Typically, this resulted in making at least two complete passes across the survey area during the time period. Upon encountering a marked bee, we netted it, placed it in a clean vial to facilitate reading the mark, and recorded the mark and geographic coordinates of capture. We conducted foraging surveys on 4 days in 2021 (between May 19 and May 25) and on 3 days in 2022 (between May 21 and May 30), and in the same areas both years.

### Data analysis

Data processing and statistical analyses were performed in R version 4.5.1 (R Core Team, 2025). Data processing used *tidyverse* (Wickham et al., 2019), and distances were calculated using the distm() function in *geosphere* (Hijmans, 2024). Kernels were fit by maximum likelihood using themle2() function in *bbmle* (Bolker & Team, 2021). Likelihood ratio tests were implemented with the Anova() function and variance inflation factors (VIFs) were calculated with the vif() function in *car* (Fox & Weisberg, 2019). Figures were created with *ggplot2* (Wickham, 2016) and *patchwork* (Pedersen, 2025). Spatial analyses were performed in QGIS v. 3.4.4‐Madeira.

For each bee recaptured at least once, we assembled chronological capture histories. We defined dispersal as successive captures at different nesting locations, that is, recaptures at the same nest were not considered dispersal. Dispersal distance was calculated as the straight‐line distance between successive nesting locations (Robusto, 1957). Foraging distance was calculated as the straight‐line distance between a capture location on flowers and most recent nesting location.

We characterized dispersal using dispersal kernels, which describe how the density of individuals changes with increasing distance from a source (Nathan et al., 2012). We fit kernels using maximum likelihood estimation to the observed frequency distributions of movement distances. We compared gamma, Weibull, and negative exponential distributions—which differ in how they describe long‐distance dispersal events—and selected the best‐fitting function using Akaike information criterion (AIC). In addition, we fit separate kernels to natal and renesting dispersal distances, and we combined their log‐likelihoods to construct a joint model representing distinct dispersal processes. We compared this joint model to a single kernel fitted to the pooled data using a likelihood ratio test.

For foraging, all three distributions received similar support (dAIC < 2; Appendix S1: Table S1), so we present Weibull foraging kernels to facilitate comparison with dispersal kernels. To evaluate whether average movement distances were sensitive to the distribution of available nesting and foraging habitat, we tested whether mean dispersal and foraging distances differed from null distributions of nesting and foraging distances, respectively (Appendix S1: Section S1).

Finally, we evaluated how four aspects of landscape composition influenced the probability of movement between two patches, that is, between two nesting sites, or between a nesting site and a foraging survey area: (1) inter‐patch distance (the straight‐line distance between patch centroids); (2) number of road crosses along the straight‐line route, (3) origin population density (the number of nests at the origin aggregation), and (4) destination patch size (the area in square meters of the destination aggregation or foraging survey area). We predicted that movement probability would decay with increasing distance and number of road crossings (Markovits et al., 2025). We had no a priori expectation for how origin population size would impact dispersal since both negative and positive density‐dependent dispersal are known from other central‐place foragers (Kim et al., 2009). And last, we expected larger patch sizes would attract dispersing bees so that dispersal would exhibit a positive relationship with patch area (Harmon‐Threatt & Anderson, 2023). We had the same expectations for foraging, except that we did not expect origin population size to be related to foraging movements, which should occur independently of conspecifics.

We modeled the probability of movement using binomial generalized linear mixed models (GLMMs), using all four landscape features as predictors and movement outcomes as successes and failures. Successes were defined as the count of movements along a particular origin–destination route, and failures were the total number of moves from that origin to all other destination patches. This approach accounts for the fact that the set of possible movement routes depends on the origin patch for each bee (following Markovits et al., 2025). Predictors were centered and scaled to improve model convergence. We initially fit models with bee ID and origin patch identity as random intercepts. In both dispersal and foraging models, the variance term associated with bee ID was effectively zero, indicating negligible among‐individual variation. Consequently, bee ID was excluded from the final models, and origin patch was retained as the only random intercept in dispersal models. For both dispersal and foraging analyses, we also initially included two‐way interactions between year and each covariate. Significance of interaction terms was evaluated using likelihood ratio tests, and nonsignificant interactions were removed to obtain final models for each movement type. Covariates were not strongly collinear (all VIFs < 2).

## RESULTS

### Dispersal

In 2021, we marked 681 unique bees and recorded 404 recaptures across 265 bees (range = 1–5 captures). In 2022, we marked 1017 bees and recorded 431 recaptures across 313 bees (range = 1–5 captures). Of these recaptures, 151 were scored as dispersal in 2021 and 198 were scored as dispersal in 2022. Females were observed using an average of 1.54 nesting locations (SD = 0.62) in 2021, and 1.62 nesting locations (SD = 0.55) in 2022 (Appendix S1: Figure S1). Since females were not necessarily marked at emergence, these values represent a conservative estimate of the number of nests constructed over a female's lifetime.

Across both years, we recorded 349 dispersal events with a median distance of 29.02 m, a mean distance of 142.3 m, and a range of 0.2 m–875 m. We recorded 13 natal dispersal events, six of which occurred within the natal aggregation (mean distance = 26.8 m) and seven of which occurred outside of the natal aggregation (mean distance = 346.3 m). We recorded 336 renesting dispersal events. Females renesting within the same aggregation dispersed an average of 13.0 m (*n* = 180), and females renesting in a different aggregation dispersed an average of 286.7 m (*n* = 156) (Appendix S1: Figure S2). A joint model describing distinct natal and renesting dispersal processes did not significantly differ than a single kernel fitted to the pooled data (likelihood ratio test: χ^2^ = 1.32, df = 1, *p* = 0.25). Notably, given large differences in sample size for natal and renesting dispersal events, our combined dataset primarily describes renesting dispersal.

**FIGURE 2:**
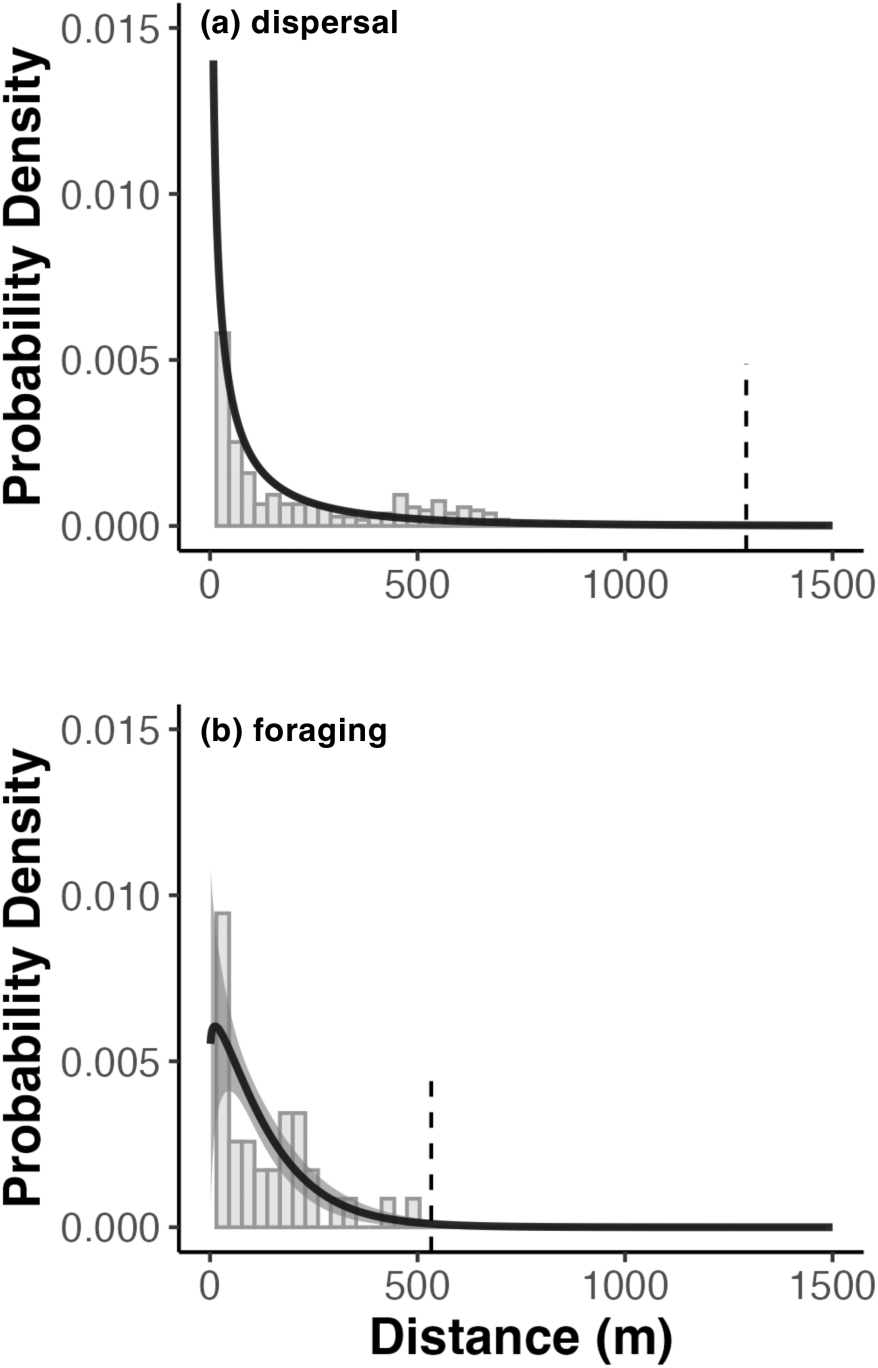
Distributions of (a) dispersal and (b) foraging distances for *Colletes validus* females. Data shown for each panel are frequency distributions of movement distances pooled across 2021 and 2022, and maximum likelihood estimates of Weibull kernels (black solid line) fit to those data. Ribbons show 95% CIs, and vertical dotted lines show 99% kernel distance to highlight differences in the distributional tails. Kernel parameters are provided in Appendix S1: Table S2.

For both 2021 and 2022 datasets, a Weibull distribution fit the dispersal data best (Appendix S1: Table S1). Dispersal kernels did not differ significantly between 2021 and 2022 (likelihood ratio test, χ^2^ = 0.53, df = 1, *p* = 0.47), so we combined results across years (see Appendix S1: Figure S3, Table S2 for annual dispersal kernels). The combined kernel was significantly fat‐tailed, indicating a greater frequency of long‐distance movements than expected under a normal distribution (Weibull kernel: shape = 0.57, 95% CI = 0.52–0.62; scale = 88.4, CI_95_ = 72.5–107.23) (Figure 2a). The mean kernel distance was 142.8 m (95% CI = 124.9–160.8); for comparison, the average of all possible inter‐nest dispersal distances was ~2–3 times higher (Appendix S1: Figure S5).

**FIGURE 3:**
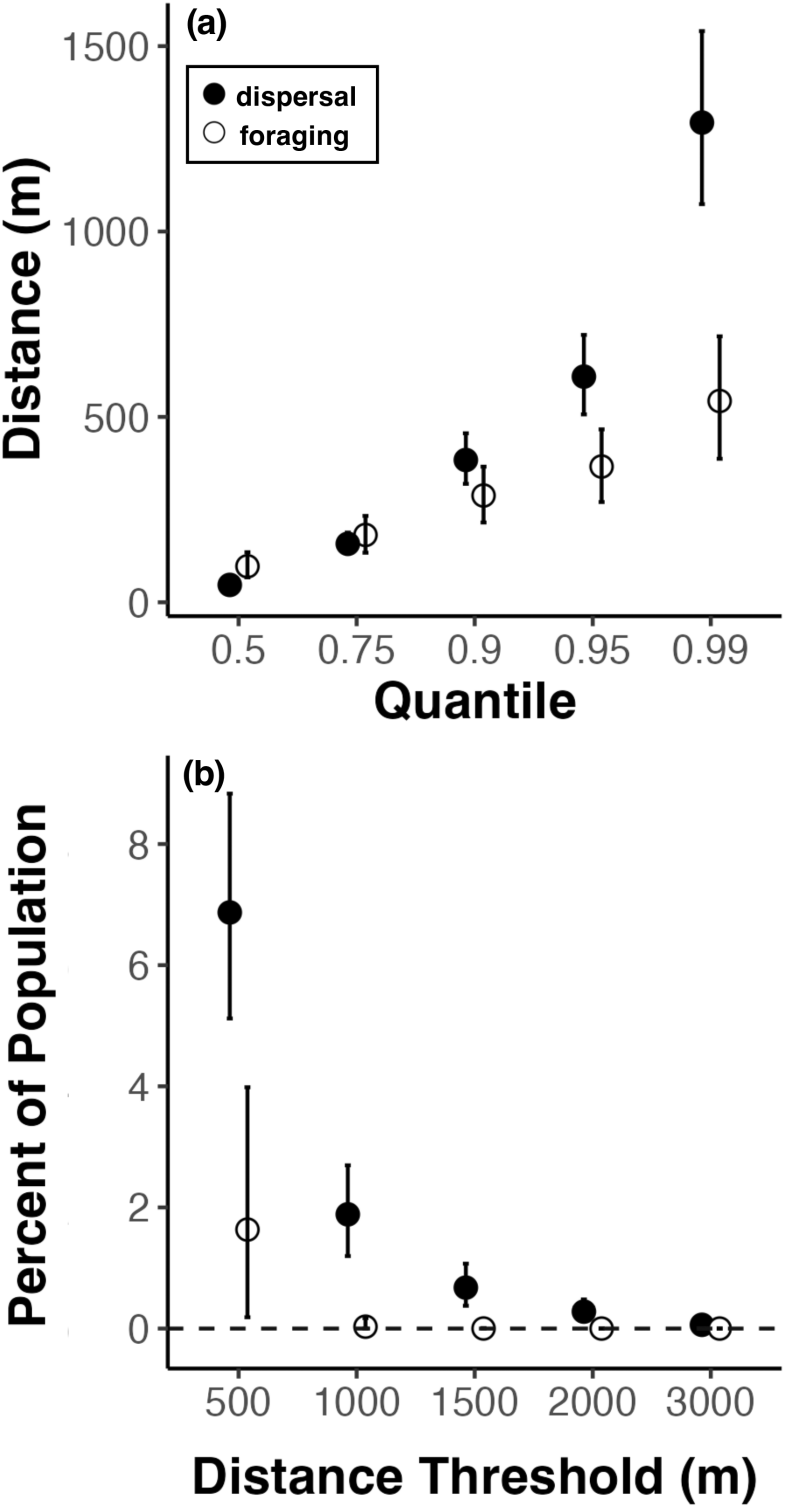
Connectivity metrics derived from movement kernels. (a) Population quantiles of Weibull foraging and dispersal kernels with bootstrapped 95% CIs. For example, values at 0.99 indicate that 99% of the population will forage within 554 m and disperse within 1291 m. (b) Movement probabilities derived from Weibull foraging and dispersal kernels. Points are the percentage of the population expected to travel beyond a threshold distance with bootstrapped 95% CIs. For example, values at 2000 m indicate that no foraging bees and 0.3% of dispersing bees can be expected to travel beyond that distance.

Dispersal exhibited long distributional tails. The 95% kernel distance (i.e., the distance below which 95% of the population is expected to disperse) was 606 m (95% CI = 507–721), and the 99% kernel distance was 1291 m (95% CI = 1074–1541) (Figure 3a). Extrapolating beyond this, approximately 0.3% and 0.05% of the population could be expected to disperse beyond 2000 and 3000 m, respectively (Figure 3b). For reference, in a population of 1698 bees (the number of marked bees in our dataset), approximately five and one migrants per generation could be expected to reach those distances, respectively.

**FIGURE 4:**
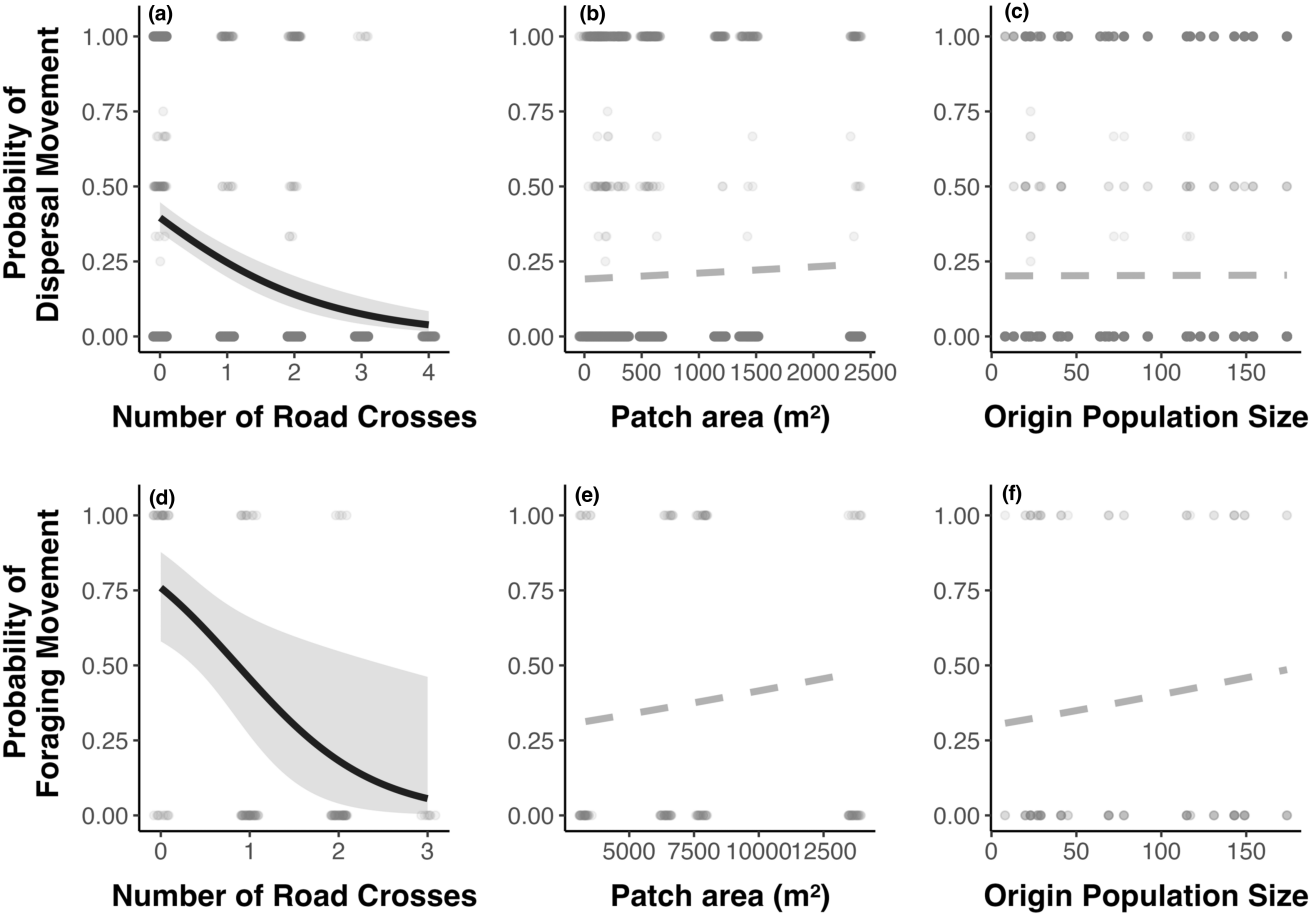
Estimated relationships between landscape features and the probability of (a–c) dispersal or (d–f) foraging. Movement probabilities were regressed against four predictors: distance among patches (among nesting aggregations or from nest to foraging survey area), number of road crosses along each straight‐line path, area of destination patch, and population size of origin patch. Each plot shows the univariate relationship between each predictor and back‐transformed probability of movement, evaluated at the mean value of the other two predictors shown and at the median dispersal or foraging distance. Solid lines with ribbons show significant relationships ±95% CIs and dotted lines show nonsignificant relationships. Points denote raw data for each pair of patches horizontally jittered to improve readability.

Dispersal propensity declined with increasing distance between patches (χ ^2^ = 398.95, df = 1, *p* < 0.001) and the number of road crossings (χ ^2^ = 44.56, df = 1, *p* < 0.001; Figure 4a; Appendix S1: Table S6). Dispersal propensity did not show a significant association with destination patch area (χ ^2^ = 1.51, df = 1, *p* = 0.22) or origin population size (χ ^2^ = 0.01, df = 1, *p* = 0.95; Figure 4c; Appendix S1: Table S6). Variation in origin patch identity explained modest variation in dispersal propensity (logit scale σ^2^ = 0.063, SD = 0.25).

### Foraging

Across both years, we observed 38 foraging events (*n*_2021_ = 18; *n*_2022_ = 20) from 38 individual bees, of which 34 were on *Vaccinium* spp. (Ericaceae) and four were on *Hudsonia ericoides* (Cistaceae). We also observed unmarked *C. validus* females foraging on fire cherry *Prunus pensylvanica* (Rosaceae), black chokeberry *Aronia melanocarpa* (Rosaceae), and bear oak *Quercus ilicifolia* (Fagaceae). Across both years, females foraged a median distance of 89.6 m, an average of 131.3 m, and a range of 6.5–491.7 m from their nests.

Foraging kernels differed significantly among years (likelihood ratio test, χ^2^ = 4.6, df = 1, *p* = 0.03); however, given the low sample sizes of each year, and to facilitate interpretation with dispersal results, we present a combined kernel (see Appendix S1: Figure S3 for annual foraging kernels). The probability of foraging declined with distance (Weibull kernel: shape = 1.08, 95% CI = 0.83–1.37; scale = 135.4, 95% CI = 97.8–184.7) (Figure 2b). The mean of the foraging kernel was 131.4 m (95% CI = 96.4–169.5 m); for comparison, the mean of the null distribution of foraging distances was ~3 times higher (Appendix S1: Figure S5).

Mean foraging kernel distance did not differ significantly from mean dispersal kernel distance, but the distributional tails of foraging were shorter than those of dispersal. The 95% and 99% foraging kernel distances were 372 m (95% CI = 270–466 m) and 554 m (95% CI = 387–717 m), respectively, both of which were significantly shorter than the 95% and 99% dispersal quantiles (Figures 2 and 3a).

Foraging propensity declined with increasing distance between nesting aggregations and foraging areas (χ ^2^ = 9.19, df = 1, *p* = 0.002) and with an increasing number of road crossings (χ ^2^ = 6.91, df = 1, *p* = 0.008; Figure 4d; Appendix S1: Table S6). Foraging propensity was not associated with destination patch area (χ ^2^ = 0.53, df = 1, *p* = 0.46) or origin population size (χ ^2^ = 0.82, df = 1, *p* = 0.36; Figure 4e,f; Appendix S1: Table S6).

## DISCUSSION

Despite the ecological significance of solitary bees, empirical data on their dispersal patterns in natural settings remain limited. By fitting statistical kernels to 2 years of mark–recapture data on *C. validus*, we found that the distribution of dispersal distances was best explained by a fat‐tailed (or leptokurtic) distribution, a pattern also exhibited by dispersal of plants (seeds) (Clark et al., 1999), birds (Fandos et al., 2023), and butterflies (Baguette, 2003). A fat‐tailed dispersal process means that while most individuals disperse short distances, a small proportion of the population travels disproportionately far. Although rare, these long‐distance movements can strongly influence ecological and evolutionary processes including genetic population structure, metapopulation persistence, and species coexistence (García & Borda‐de‐Água, 2017; Nathan, 2001; Sullivan et al., 2021). For *C. validus*, the mean dispersal distance (143 m) was an order of magnitude shorter than the tails of the distribution, with the farthest dispersers expected to move nearly 3 km (Figure 3b), implying that solitary bee populations may be connected by infrequent but distant dispersal movements, even when most nesting occurs locally.

Past work has noted contrasting findings regarding the dispersal ecology of solitary bees: Some studies report low genetic differentiation among populations, consistent with high dispersive capacity (Davis et al., 2010; Dellicour et al., 2014), whereas others report philopatry, suggesting a reluctance to disperse far distances (Antonini et al., 2000; Steffan‐Dewenter & Schiele, 2004; Yanega, 1990). Our study reconciles this apparent contradiction by demonstrating that breeding‐site philopatry and long‐distance dispersal occur within the same system (Ballare & Jha, 2021; López‐Uribe et al., 2015; Steffan‐Dewenter & Schiele, 2004). This pattern held for both natal and renesting dispersal, with roughly half of movements in each process occurring between distinct nesting aggregations. Taken together, our results demonstrate that a high frequency of philopatry does not necessarily preclude population connectivity.

These findings are also consistent with the natural history of *C. validus*, which nests in a naturally patchy, disturbance‐dependent landscape. Philopatry allows renesting bees to take advantage of high‐quality nesting sites locally, while long‐distance dispersal allows for colonization of newly disturbed sites. Indeed, some aggregations during our study were occupied perennially, whereas others were occupied ephemerally, only colonized by dispersing bees partway through the nesting season. Interestingly, aggregations colonized early in the nesting season tended to persist among years, whereas late‐colonized aggregations tended not to be occupied the following year, possibly due to only partial nests being constructed late in the growing season. These observations with *C. validus* help to explain similar metapopulation dynamics from other ground‐nesting solitary bees (Franzén & Nilsson, 2010) and, more broadly, support the idea that animal dispersal comprises multiple ecologically distinct processes (Kesler et al., 2010).

Many ecological processes such as responses to landscape change, range shifts, and patch colonization are inherently spatial and therefore linked to dispersal. Most past studies on bee movement, however, have focused on foraging, which may differ in spatial scale from dispersal since the two movement types can be shaped by different resource distributions and occur at different frequencies throughout the life cycle. Despite the fact that our foraging distance estimates were based on only 38 bees, *C. validus* foraging distances aligned with predictions from published bee body size–foraging distance relationships (Gathmann & Tscharntke, 2002; Kendall et al., 2022); we estimated a 99% foraging kernel distance of *C. validus* of 554 m, and these models—evaluated at a body length of 14 mm and mean body mass of 0.029 g, respectively—predict maximum foraging distances for *C. validus* of 533 m and 578 m. Comparing foraging to dispersal revealed a key difference between the two movement types: Mean movement distances were similar, but long‐distance dispersal was twice as great as long‐distance foraging (Figure 3a). This pattern in solitary bees is consistent with the general pattern from other central‐place foragers that dispersal occurs over greater spatial scales than foraging (Fernández‐Chacón et al., 2013; Mola & Williams, 2025). Consequently, these results underscore the importance of distinguishing between foraging and dispersal movements when evaluating population connectivity of central‐place foragers.

One feature of our study is that we measured movement of wild bees throughout an existing network of habitat patches, providing realized dispersal and foraging estimates in a natural landscape. At the same time, observed movement patterns may also have been shaped by the particular landscape context in which our study took place (Fahrig, 2007; Herrera et al., 2016). After controlling for the distance between patches, we found that dispersal and foraging movements were significantly less likely to occur among patches separated by a greater number of road crossings, consistent with past mark–recapture studies on foraging bees (Markovits et al., 2025; Roper & Youngsteadt, 2025) (Figure 4a,d). This finding offers an empirical mechanism behind previously documented patterns of reduced gene flow of wild bees in anthropogenic landscapes (Jha & Kremen, 2013) and demonstrates that ecologically distinct movements can exhibit consistent responses to landscape features. In contrast to our expectation, however, dispersal movements in our system were not sensitive to patch area, a finding which agrees with one past study in solitary bees (Franzén & Nilsson, 2010). How habitat heterogeneity and landscape fragmentation come together to influence insect movement and population viability in both natural and urban settings is a compelling area for future work.

In recent years, interest in solitary bee conservation has surged in response to widespread declines. Restoration of spatial population structure is a central goal in the conservation of threatened taxa, which requires not only enhancing habitat quality within patches, but also facilitating dispersal among patches (Schultz et al., 2018). Although realized dispersal distances likely vary among bee taxa and environmental contexts, our study provides initial quantitative benchmarks for inter‐patch distances to facilitate patch colonization by solitary bees (Figure 3). For *C. validus*, patches separated by ~150 m (mean dispersal distance) will receive the greatest number of dispersing individuals, but patches separated between 1 and 3 km will still receive some level of dispersal, broadly consistent with dispersal estimates for queen bumble bees of 1.2–3.3 km during the solitary nest‐founding phase (Bowers, 1985; Mola et al., 2020). For a population of 1698 solitary bees (the number marked in our study), patches separated by 1 km will receive ~30 dispersing females within a nesting season, whereas patches separated by 3 km will receive ~1 female. One rule of thumb suggests that one migrant per generation can maintain genetic connectivity among populations (Mills & Allendorf, 1996), meaning that patches 3 km apart may be an upper limit for inter‐patch distances when genetic connectivity is the goal. In addition, because our study focused on females, our estimates may represent a conservative view of connectivity. In many central‐place foragers, dispersal is male‐biased (Greenwood, 1980) and in solitary bees, males—which are not tied to a nesting location—may disperse farther and more frequently than females (López‐Uribe et al., 2015). If *C. validus* exhibits male‐biased dispersal, populations could be connected by gene flow over even greater distances than those inferred from female dispersal movements alone. Future work comparing male and female dispersal behavior in solitary bees could help to contextualize patterns of genetic connectivity across the landscape.

Generalizing these results with *C. validus* to other bee taxa will require additional studies that empirically measure dispersal kernels, and we hope that our study offers a framework for how similar movement data could be gathered. Ideal solitary bee species for studying movement processes will have traits conducive to mark–recapture such as being identifiable “on the wing,” having a large enough body size to apply individual markings, and nesting in large aggregations (Dorian et al., 2024). Regardless of system, we emphasize the potential of small, short‐lived insects for advancing our understanding of the dispersal ecology of central‐place foragers.

## AUTHOR CONTRIBUTIONS

Nicholas N. Dorian and Elizabeth E. Crone jointly conceived of these ideas. Nicholas N. Dorian performed field work and analyzed data with guidance from Elizabeth E. Crone. Nicholas N. Dorian wrote the first draft of the manuscript. Nicholas N. Dorian and Elizabeth E. Crone edited all parts of the manuscript and agreed to the content.

## CONFLICT OF INTEREST STATEMENT

The authors declare no conflicts of interest.

## Supporting information


Appendix S1.

## Data Availability

Data and code (Dorian, 2026) are available in the Open Science Framework repository at https://doi.org/10.17605/OSF.IO/VGK3W.
