# Supplementary material for: Fat‐tailed dispersal shapes connectivity in a solitary bee
Source: Ecology. 2026 Jul 22;107(7):e70437. doi: 10.1002/ecy.70437 (PMC13389559; doi:10.1002/ecy.70437)
Supplement: Supplementary file 1 — Appendix S1. [file ECY-107-e70437-s001.pdf]

Appendix S1

Fat-tailed dispersal shapes connectivity in a solitary bee

Nicholas N. Dorian & Elizabeth E. Crone

Ecology

Table S1. Comparison of model fit for three different kernel shapes. Weibull kernels were selected to facilitate quantitative comparison of foraging and dispersal movement distributions.

**Movement type**	**Year**	**dAIC Exponential** (df = 1)	**dAIC Gamma** (df = 2)	**dAIC Weibull** (df = 2)
Dispersal	Pooled	231.8	14.3	0.0
Dispersal	2021	85.6	5.2	0.0
Dispersal	2022	119.6	8.1	0.0
Foraging	Pooled	0.0	1.8	1.8
Foraging	2021	0.0	0.3	0.3
Foraging	2022	0.0	2.5	2.5

Table S2. Weibull kernel parameters ± 83.4% confidence intervals. Mean values are distances in meters and are a function of both shape and scale parameters.

**Movement type**	**Year**	**Shape**[1]	**Scale**[1]	**Mean**
Foraging	2021	1.34 (0.97-1.71)	185.24 (140.4-230.0)	170.1 (130.6-209.4)
Foraging	2022	0.99 (0.80-1.18)	96.56 (65.1-128.0)	96.8 (67.9-125.9)
Foraging	Both	1.08 (0.9-1.29)	135.5 (108.1-168.4)	131.4 (105.5-157.3)
Dispersal	2021	0.59 (0.54-0.64)	93.01 (75.74-113.75)	143.5 (124.3-162.8)
Dispersal	2022	0.56 (0.53-0.58)	84.81 (68.9-100.7)	142.2 (118.4-165.9)
Dispersal	Both	0.57 (0.54-0.59)	88.26 (70.3-106.2)	142.8 (117-5-168.2)

[1]A Weibull distribution consists of two parameters, a shape parameter which describes the skew of the distribution, and a scale parameter which is related to the mean. A shape parameter less than one indicates a fat-tailed, or leptokurtic, distribution whereas a shape parameter greater than one indicates a narrow-tailed, or platykurtic, distribution. Ecologically, in leptokurtic distributions long dispersal events are more common than expected under exponential decay.

Table S3. Log likelihoods of models fit to different movement types among years. N represents the sample size for each movement type.

**Movement type**	**Year**	**Model log likelihood**	**N**
Testing for differences between natal vs. renesting movements in each year			
Natal	2021	-58.796	10
Re-nesting	2021	-797.536	141
Both	2021	-856.424	151
Natal	2022	-19.658	3
Re-nesting	2022	-1084.229	195
Both	2022	-1105.658	198
Natal	2021 + 2022	-79.62	13
Re-nesting	2021 + 2022	-1882.08	336
Both	2021 + 2022	-1962.35	349
Testing for differences between dispersal kernels among years			
Dispersal	2021	-856.424	151
Dispersal	2022	-1105.658	198
Dispersal	2021 + 2022	-1962.345	349
Testing for differences between foraging kernels among years			
Foraging	2021	-109.28	18
Foraging	2022	-111.47	20
Foraging	2021 + 2022	-223.14	38

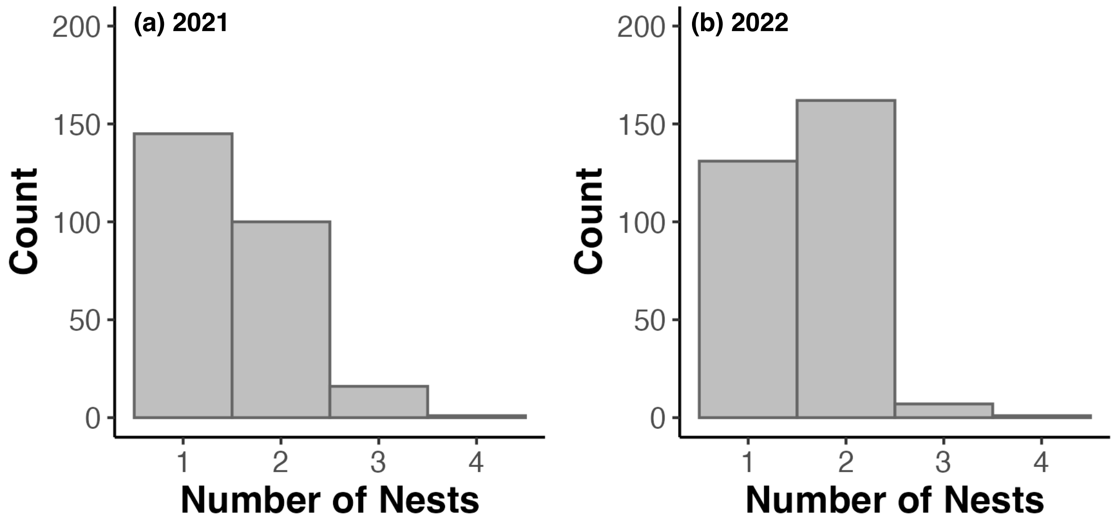

Figure S1. Number of nests built by female *Colletes validus* in a) 2021 and b) 2022 based on recapture of individually marked bees.

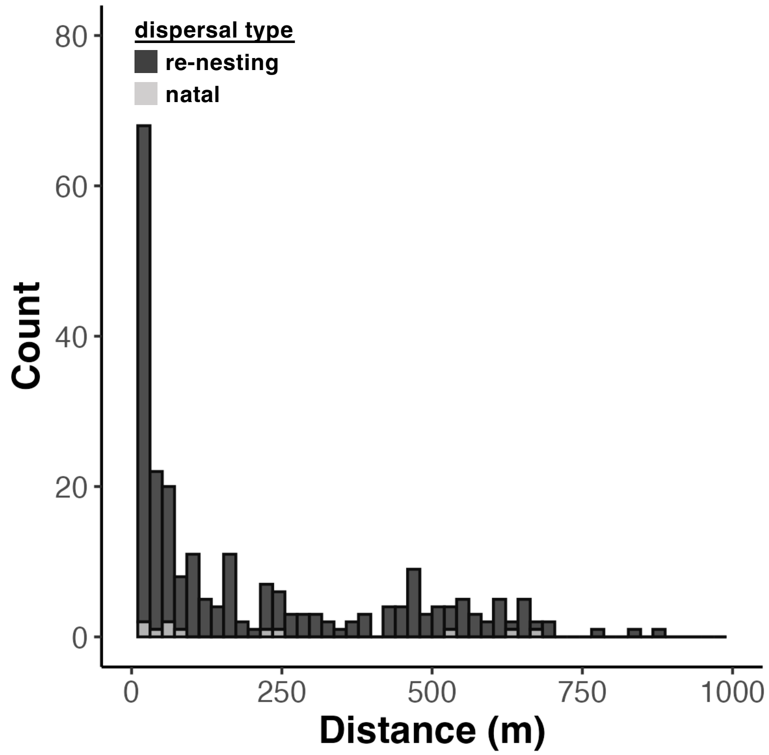

Figure S2. Natal (n = 13) and re-nesting (n = 336) dispersal distances for 2021 and 2022 combined.

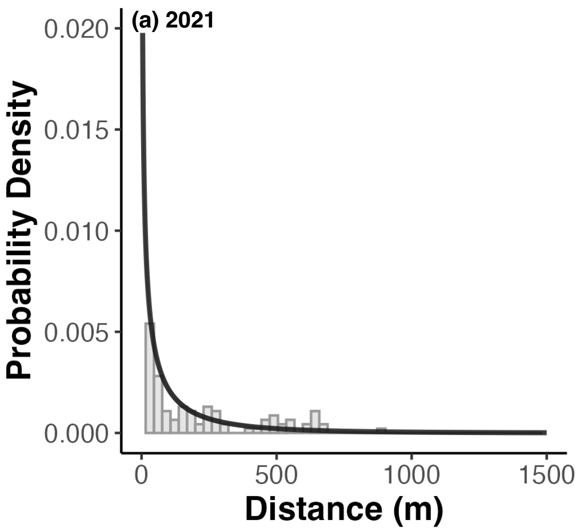

Figure S3. Weibull kernels fit to a) 2021 and b) 2022 dispersal data. Dispersal distances are straight-line distances between two unique nests, two unique aggregations, or an emergence tent to a nest or aggregation. Both natal and re-nesting dispersal distances are shown. Kernel parameters are provided in Table S2.

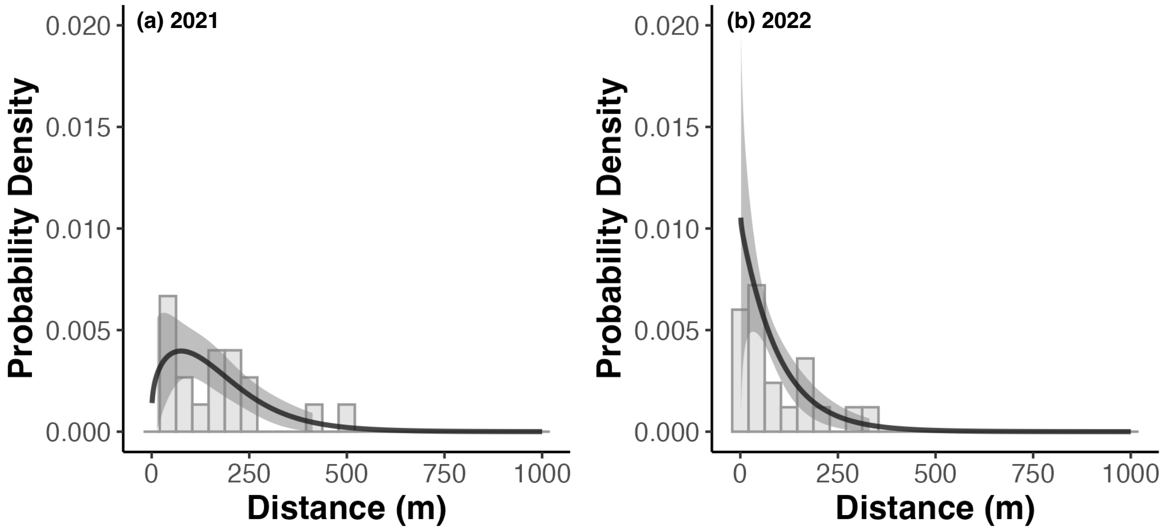

Figure S4. Weibull foraging kernels fit to a) 2021 and b) 2022 movement data. Foraging distances are straight-line distances between a nest and flower; ribbons show 95% confidence intervals. Kernel parameters are provided in Table S2.

Table S4. Distance (m) within which each quantile of the population is expected to disperse. For example, 99% of the population is expected to disperse within 1291.1 m; conversely the top 1% of dispersers will migrate at or beyond that distance.

**movement type**	**quantile**	**mean**	**lci**	**uci**
dispersal	0.50	46.4	37.7	57.5
dispersal	0.75	156.6	130.7	187.9
dispersal	0.90	382.1	319.5	455.8
dispersal	0.95	606.6	506.8	721.2
dispersal	0.99	1291.1	1073.8	1540.5
foraging	0.50	96.5	67.3	135.1
foraging	0.75	183.0	133.8	232.9
foraging	0.90	292.3	215.3	365.8
foraging	0.95	372.6	270.4	466.4
foraging	0.99	554.0	387.1	717.2

Table S5. Percentage of the population expected to disperse beyond a particular distance. For example, 0.06% of the population is expected to disperse at or beyond 3000m. In a population of 1698 bees (the number of marked bees in our study) that translates to ~1 individual.

**movement type**	**distance**	**mean (%)**	**lci (%)**	**uci (%)**
dispersal	500	6.83	5.12	8.83
dispersal	1000	1.86	1.20	2.69
dispersal	1500	0.66	0.38	1.07
dispersal	2000	0.27	0.14	0.48
dispersal	3000	0.06	0.02	0.12
foraging	500	1.60	0.19	3.98
foraging	1000	0.02	0.00	0.17
foraging	1500	0.0001	0.00	0.01
foraging	2000	0.00	0.00	0.00
foraging	3000	0.00	0.00	0.00

Table S6. Covariate estimates and marginal hypothesis test results for final multiple regression model exploring effects of landscape features on solitary bee dispersal and foraging. All predictors were scaled, i.e. centered on the mean of each predictor and divided by the standard deviation.

**movement type**	**predictor**	**estimate**	**std. err**	χ^2	**df**	**p**
dispersal	distance	-3.08	0.204	227.80	1	**<0.001**
	year	0.10	0.366	0.07	1	0.788
	distance x year	0.36	0.252	2.06	1	0.151
	patch area	0.07	0.060	1.51	1	0.219
	number road crossings	-0.68	0.10	44.56	1	**<0.001**
	origin population size	0.004	0.076	0.01	1	0.950
foraging	distance	-1.18	0.430	9.19	1	**<0.01**
	destination patch area	0.25	0.345	0.53	1	0.464
	number road crossings	-1.12	0.448	6.91	1	**<0.01**
	origin population size	0.24	0.272	0.82	1	0.36

SECTION S1: SUPPLEMENTARY ANALYSIS

We tested whether mean dispersal and foraging distances differed from random distributions. For dispersal, we first calculated pairwise distances between all nests. Then, we drew 1000 random samples from this distribution, each equal to the length of our observed dataset, and calculated the mean and standard error of those samples. We determined whether the mean kernel dispersal distance was significantly different from the mean of the random dispersal distribution by assessing whether the 83.4% confidence intervals overlapped (MacGregor-Fors and Payton 2013). Comparing the overlap of 83.4% confidence intervals allows us to assess statistical significance from interval overlap at $\alpha = 0.05$ (MacGregor-Fors and Payton 2013). The observed average dispersal kernel distance (see main text) was 142.8m ($CI_{83.4}$ = 125.0-160.7), which was significantly lower than the average of inter-nest distances (422.9m, $CI_{83.4}$ = 403.3-441.6m; Figure S5a).

We also considered whether the observed distribution of foraging distances was significantly different from a random distribution. To do so, we generated 1000 random points within the boundaries of all four foraging survey areas combined (fig. A1). Then, we estimated the distance between the 1000 random points and each unique nest location in both 2021 and 2022. We calculated the mean of this null distribution of random points and bootstrapped the standard error. We determined whether the observed distribution of foraging movements was significantly different from null by assessing whether the 83.4% confidence intervals of the mean of the two distributions overlapped (MacGregor-Fors and Payton 2013). On average, females foraged significantly closer to their nest (mean of 131.4m, $CI_{83.4}$ = 105.5-157.3; see main text) than the mean of a distribution of random foraging distances (406.0m, $CI_{83.4}$ = 356.5-453.9m; Figure S5b).

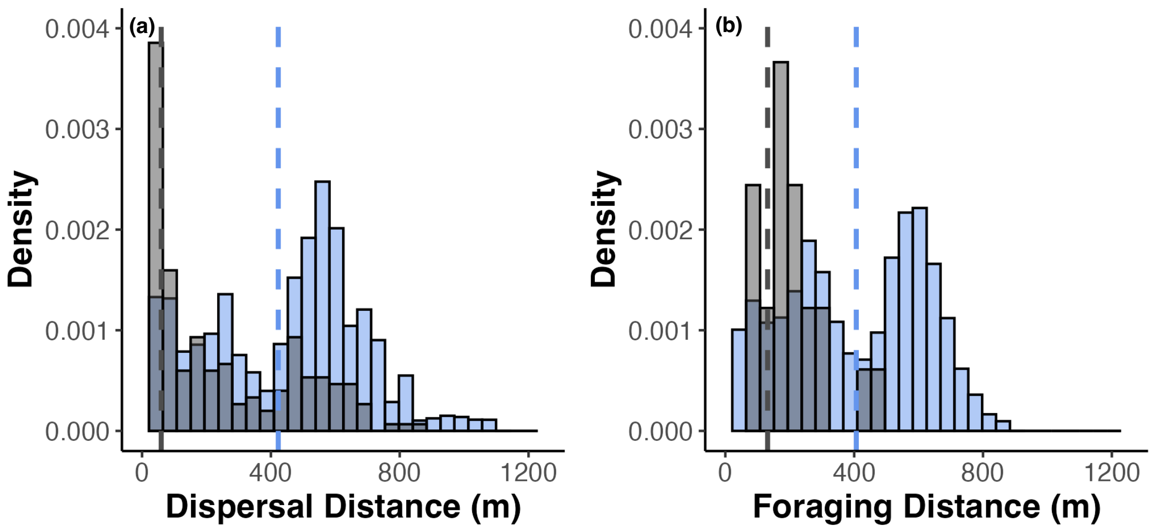

Figure S5. Distribution of observed (gray) vs. null (light blue) movement distances. (a) A null distribution of dispersal distances was estimated from the pairwise distances among all observed nests. (b) A null distribution of foraging distances was estimated from the distribution of nest locations to 1000 random points within the four foraging survey areas.

REFERENCES

Harmon-Threatt AN, Anderson NL (2023) Bee movement between natural fragments is rare despite differences in species, patch, and matrix variables. Landsc Ecol 38:2519–2531. https://doi.org/10.1007/s10980-023-01719-6

MacGregor-Fors I, Payton ME (2013) Contrasting Diversity Values: Statistical Inferences Based on Overlapping Confidence Intervals. PLOS ONE 8:e56794. https://doi.org/10.1371/journal.pone.0056794

Markovits CM, Dorian NN, Crone EE (2025) Roads are partial barriers to foraging solitary bees in an urban landscape. Oecologia 207:7. https://doi.org/10.1007/s00442-024-05652-6
